# Preparation of a Zirconium-89 Labeled Clickable DOTA Complex and Its Antibody Conjugate

**DOI:** 10.3390/ph17040480

**Published:** 2024-04-09

**Authors:** Falguni Basuli, Olga Vasalatiy, Jianfeng Shi, Kelly C. Lane, Freddy E. Escorcia, Rolf E. Swenson

**Affiliations:** 1 Chemistry and Synthesis Center, National Heart, Lung and Blood Institute, Bethesda, MD 20892, USA; vasalatiyo@nhlbi.nih.gov (O.V.); jianfeng.shi@nih.gov (J.S.); kclane013@gmail.com (K.C.L.); rolf.swenson@nih.gov (R.E.S.); 2 Molecular Imaging Branch, Center for Cancer Research, National Cancer Institute, Bethesda, MD 20892, USA; freddy.escorcia@nih.gov; 3 Radiation Oncology Branch, Center for Cancer Research, National Cancer Institute, Bethesda, MD 20892, USA

**Keywords:** DOTA, BCN-DOTA-GA, PAN, DFO, DFO*, stability

## Abstract

Desferrioxamine B (DFO) is the clinical standard chelator for preparing zirconium-89 labeled antibodies. In the current study, the stabilities of a zirconium-89 labeled panitumumab (PAN; Vectibix^®^) with three different chelators (DFO, DFO*, and DOTA) were compared. PAN is an anti-HER1/EGFR monoclonal antibody approved by the FDA for the treatment of HER1-expressing colorectal cancers and was used as the model antibody for this study. DFO/DFO* conjugates of PAN were directly radiolabeled with zirconium-89 at room temperature to produce [^89^Zr]Zr-DFO/DFO*-PAN conjugates following a well-established procedure. A zirconium-89 labeled DOTA-PAN conjugate was prepared by an indirect radiolabeling method. A cyclooctyne-linked DOTA chelator (BCN-DOTA-GA) was first radiolabeled with zirconium-89 at 90 °C under a two-step basic pH adjustment method followed by conjugation with PAN-tetrazene at 37 °C to produce a labeled conjugate, BCN-[^89^Zr]Zr-DOTA-GA-PAN. High reproducibility of the radiolabeling was observed via this two-step basic pH adjustment. The overall radiochemical yield was 40–50% (n = 12, decay uncorrected) with a radiochemical purity of >95% in 2 h synthesis time. All three conjugates were stable in whole human serum for up to 7 days at 37 °C. The kinetic inertness of the conjugates was assessed against the EDTA challenge. BCN-[^89^Zr]Zr-DOTA-GA-PAN exhibited excellent inertness followed by [^89^Zr]Zr-DFO*-PAN. [^89^Zr]Zr-DFO-PAN displayed the lowest level of inertness.

## 1. Introduction

Positron emission tomography (PET) is favorable among the clinically established non-invasive imaging techniques due to its unique properties such as excellent sensitivity, spatial resolution, and accurate quantification of small tumor lesions [1,2,3]. The functional insights acquired via PET imaging enable an earlier diagnosis and clear characterization of the disease state, which is crucial for providing a reliable prognosis and guiding effective therapeutic strategies [1]. Many radiolabeled peptides and protein-based drugs have been developed for diagnosis and therapy [1,4,5,6,7,8]. Gallium-68 and fluorine-18 are commonly used PET radionuclides for the production of low-molecular-weight tracers; however, large-molecular-weight proteins and antibodies require longer-lived radionuclides due to their slower in vivo pharmacokinetics and long blood pool residence time. Among the longer-lived PET isotopes, zirconium-89 (t_1/2_ = 78.4 h; E*_β_*^+^_(mean)_ = 396 keV; E_max_ = 897 keV) has been widely used to radiolabel antibodies due to its favorable decay characteristics and the excellent match of the zirconium-89 half-life with the time an antibody needs to reach the target [9,10,11,12,13]. In addition, the half-life of zirconium-89 is suitable to produce a companion diagnostic imaging agent for actinium-225 (t_1/2_ = 9.9 days)- and thorium-227 (t_1/2_ = 18.7 days) labeled therapeutic radiopharmaceuticals [14,15]. Moreover, zirconium-89 can be produced in large quantities via the proton irradiation of the monoisotopic yttrium (^89^Y) target on a low-energy medical cyclotron and can be efficiently separated from the target [16].

**Figure 1:**
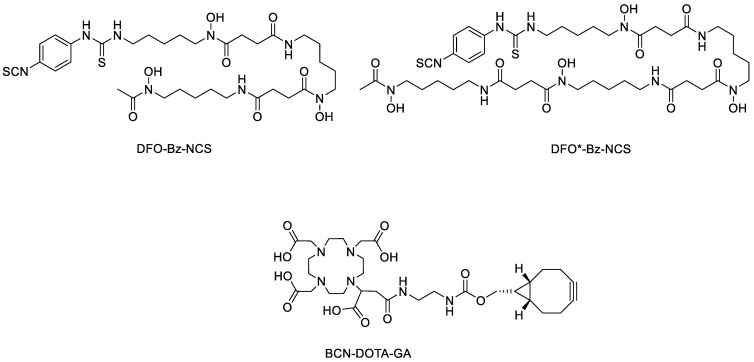
Structures of the target chelators DFO-Bz-NCS, DFO*-Bz-NCS, and BCN-DOTA-GA.

Typically, when incorporating a radiometal for imaging or targeted radionuclide therapy, it is crucial to utilize an appropriate chelator with accessible Lewis basic donor groups for the effective binding and stabilization of the radiometal ion. Additionally, the chelator should contain a reactive functional group for bioconjugation under mild conditions. A hydroxamate-based natural siderophore chelator, desferrioxamine B (DFO, Figure 1), is the gold standard in preclinical and clinical studies for incorporating zirconium-89 into the biomolecules of interest [10,17,18,19,20,21]. However, several investigations have pointed out that the limited kinetic inertness of [^89^Zr]Zr–DFO complexes leads to the liberation of free zirconium ([^89^Zr]Zr^4+^) [22,23,24,25,26]. Free [^89^Zr]Zr^4+^ accumulates into the bones and joints, which compromises the quality of images, and it increases the radiation dose to hematopoietic bone marrow [24,27,28,29]. The observed instability of the [^89^Zr]Zr–DFO complex is partially a consequence of the mismatch between the zirconium ion’s coordination number of eight and DFO’s hexadentate chelation, which leaves two coordination sites open to interact with other substrates [30,31,32]. Similarly, DFO may not be a suitable chelator to develop a theranostic (therapeutic and diagnostic) agent for therapeutic radiometals (e.g., actinium-225 or thorium-227), which typically form stable eight-coordinate species as well. This emphasizes the need for superior chelators with enhanced stability, which could potentially reduce the uptake of radionuclides in the bone and streamline the development of theranostic agents.

Several new chelators (≥8 coordination sites) that can saturate the zirconium ion coordination sphere, such as DFO* (Figure 1), oxoDFO*, HOPO, CTH-36, DFO-Em, DFO-Km, DFO2, DFO2p, etc., have been developed and investigated [24,26,33,34,35,36,37,38,39,40,41]. Many of these complexes have demonstrated superior in vivo stability compared to DFO. Patra et al. developed the first octadentate bifunctional chelating agent, DFO*, for zirconium-89 labeling [24]. Salih et al. designed and synthesized a glutamic acid-based octadentate chelator named DFO-Em [38]. However, the attempted synthesis of the bifunctional version of the DFO-Em via N-hydroxysuccinimide (NHS) ester activation of the glutamic acid side chain for conjugation to biomolecules was unsuccessful. Therefore, they replaced the glutamic acid linker in DFO-Em with lysine to prepare another chelator, DFO-Km [37]. One of the key highlights of DFO-Em and DFO-Km ligands is in their modular amino acid linker system that can be swapped with other amino acid linkers or chelating moieties, thereby enabling the synthesis of wide varieties of derivatives. Recently, Damerow et al. compared the relative stability of [^89^Zr]Zr-DFO-, [^89^Zr]Zr-CTH-36-, [^89^Zr]Zr-3,4,3-(LI-1,2-HOPO), and [^89^Zr]Zr-DFO*-c(RGDfK) complexes against challenge with ethylenediaminetetraacetic acid (EDTA) [42]. A rapid trans chelation was observed for the [^89^Zr]Zr-DFO complex, which indicates a low kinetic inertness of this conjugate. The [^89^Zr]Zr-CTH-36-c(RGDfK) complex demonstrated unexpected susceptibility to the challenge. However, [^89^Zr]Zr-3,4,3-(LI-1,2-HOPO) and [^89^Zr]Zr-DFO*-c(RGDfK) were highly stable. 

A tetraazamacrocycle ligand, 1,4,7,10-tetraazacyclododecane-1,4,7,10-tetraacetic acid (DOTA), is a well-known chelator in radiopharmaceutical applications as it can readily form stable complexes with numerous radiometals, such as [^68^Ga]Ga, [^64^Cu]Cu, [^90^Y]Y, [^117^Lu]Lu, [^225^Ac]Ac, and [^227^Th]Th [14,15,43,44,45,46]. Moreover, the same DOTA-functionalized molecule can be radiolabeled with either diagnostic or therapeutic radiometals to form theranostic radiopharmaceuticals, which will allow for the accurate determination of the radiation dose before treatment as well as excellent monitoring of treatment effectiveness [47,48]. In 2017, Pandya et al. reported the structural characterization of Zr–DOTA using single-crystal X-ray diffraction and the use of DOTA as [^89^Zr]Zr-chelators [49]. The crystal structure reveals a saturated coordination sphere around the Zr^4+^ ion. Additionally, [^89^Zr]Zr-DOTA demonstrated remarkable in vivo stability. Prive et al. further optimized the reaction conditions to successfully prepare zirconium-89 labeled DOTA-PSMA-617 and DOTAGA-PSMA-I&T and they demonstrated its first clinical application in a human patient [50]. However, Damerow et al. observed no labeling of the DOTA-GA-modified peptide even after applying harsh conditions (99 °C for several hours). Herein, the synthesis of BCN-[^89^Zr]Zr-DOTA-GA under two-step pH adjustments at basic conditions is presented. This zirconium-89 labeled BCN prosthetic group was used for labeling PAN via a well-known click approach to prepare BCN-[^89^Zr]Zr-DOTA-GA-PAN and the stability of this complex was compared with [^89^Zr]DFO/DFO*-PAN conjugates. The BCN-[^89^Zr]Zr-DOTA-GA-PAN conjugates showed excellent stability in whole human serum and an EDTA challenge study for up to 7 days. 

## 2. Results and Discussion

Although DOTA is commonly used as a chelator to prepare various radiopharmaceuticals with radiometals, its application with zirconium-89 remains relatively unexplored. In 2017, Pandya et al. reported the successful synthesis of zirconium-89 labeled DOTA using [^89^Zr]ZrCl_4_ [49]. Labeling of DOTA with [^89^Zr]Zr-oxalate (commonly used for zirconium-89 labeling) resulted in poor radiochemical conversion. A quantitative formation of a radiolabeled DOTA complex, [^89^Zr]Zr-DOTA, was observed by incubating 10 µg of DOTA with an aliquot of [^89^Zr]ZrCl_4_ (1.1 mCi, 40.7 MBq) at 90 ^°^C in HEPES buffer (pH 7.2) for 1 h. To test the feasibility of the preparation of a radiolabeled p-SCN-Bz-DOTA bifunctional chelator, the non-radioactive chelation chemistry was explored first. A non-radioactive zirconium complex of p-SCN-Bz-DOTA was successfully prepared by reacting ZrCl_4_ with p-SCN-Bz-DOTA at 80 ^°^C for 2 h in anhydrous dimethyl sulfoxide (DMSO) in the presence of triethylamine [51]. However, under the same condition, the zirconium-89 radiolabeling reaction proceeded without the formation of any desired labeled product. The aqueous basic radiolabeling condition resulted in the decomposition of the chelator. 

**Scheme 1:**
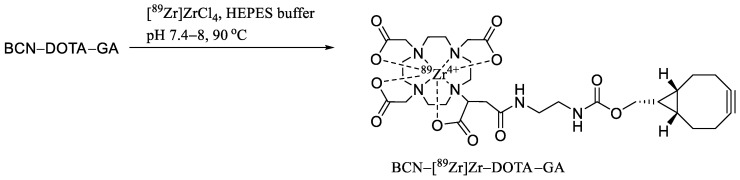
Preparation of BCN-[^89^Zr]Zr-DOTA-GA.

Therefore, the labeling of another ligand, DOTA-GA, functionalized with BCN (Figure 1, Scheme 1), a cyclooctyne for copper-free click chemistry, as it is stable in aqueous basic conditions, was explored. The radiochemical conversion was first tested with [^89^Zr]Zr-oxalate and the result monitored by analytical high-performance liquid chromatography (HPLC). HPLC analysis revealed the formation of no new peaks, and the same results have also been reported by other researchers [42]. The radiolabeling of BCN-DOTA-GA was then tested under various conditions (Table 1) using [^89^Zr]ZrCl_4_. The radiochemical conversion (RCC) was monitored by HPLC and radio thin-layer chromatography (TLC). Less than 20% conversion was observed with 10 µg of chelator and 1 mCi of [^89^Zr]ZrCl_4_ at 80 °C for 1 h. No improvement in the RCC was observed by increasing the temperature to 90 °C with the same amount of the chelator and zirconium-89. 

**Figure 2:**
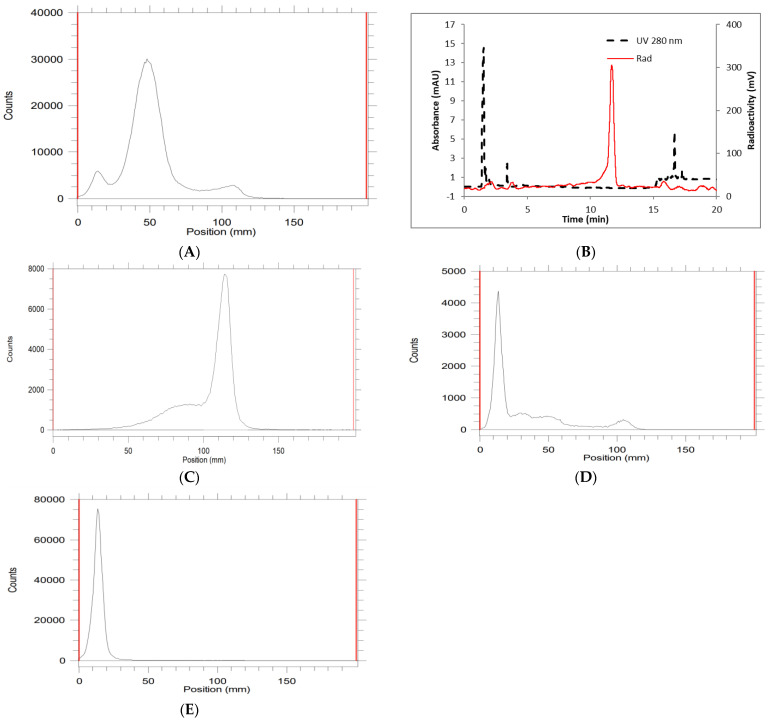
Representative radiochemical conversion to BCN-[^89^Zr]Zr-DOTA-GA in two-step pH adjustment (**A**) radio TLC; (**B**) analytical HPLC. Radio TLC of (**C**); [^89^Zr]Zr-EDTA; (**D**) BCN-[^89^Zr]Zr-DOTA-GA reaction starting at high pH; (**E**) [^89^Zr]ZrCl_4_ heated at high pH (~8). TLC condition: iTLC-SG, developer, 0.1 M EDTA pH 7; HPLC condition: Agilent XDB C-18 (4.6 × 150 mm, 5 µ); eluent, 0–45% B in 13 min, 45–90% B from 13–14 min, 90% B until 19 min, A = 0.05% TFA in water, B = 0.05% TFA in acetonitrile, flow rate 1 mL/min; black line UV detector, red line radio detector. Retention time = 11.6 min.

However, an increasing amount of the chelator (30 µg) demonstrated a nearly quantitative RCC (Table 1, entry 4) at 90 °C. A higher amount of zirconium-89 (2–4 mCi) with the same amount of chelator (30 µg) at the same temperature (90 °C) resulted in a decreased RCC (entry 5–7). In order to balance the RCC and the molar activity, 30 µg ligand, 3 mCi zirconium-89, and 90 °C were chosen as the optimal conditions. After incubation for 1 h, an aliquot of the reaction mixture was mixed with EDTA and analyzed by radio TLC and analytical HPLC. A representative radio TLC (developer, 0.1 M EDTA) and HPLC (eluent, acetonitrile in water) are depicted in Figure 2A,B. The major radio TLC peak at 50 mm represents BCN-[^89^Zr]Zr-DOTA-GA. The minor radio TLC peak at 100 mm matches with the radio TLC peak for [^89^Zr]Zr-EDTA (Figure 2C) and the other minor peak at 10 mm could be the hydroxide species of zirconium-89. It is important to mention here that a gradual increase in the pH is necessary for a successful radiolabeling reaction. The initial pH of the reaction mixture was ~7.2 after mixing zirconium-89 and the BCN-DOTA-GA chelator. The reaction mixture was heated at 90 °C for 30 min and the pH was adjusted to ~7.6 followed by heating for an additional 30 min at the same temperature. If the pH of the reaction was adjusted to ~7.6 at the beginning of the reaction, then the formation of a trace amount of BCN-[^89^Zr]Zr-DOTA-GA was observed by TLC analysis (Figure 2D). A major radio TLC peak at 10 mm was observed. This could be due to the hydrolysis of [^89^Zr]ZrCl_4_ to inert zirconium hydroxide as reported in the literature [42]. This peak matches with the radio TLC of the [^89^Zr]ZrCl_4_ after heating at 90 °C for 30 min (Figure 2E) at pH ~8. The successful radiolabeling with a two-step pH adjustment at the acidic conditions was reported by Privé et al. in 2021 [50]. The PSMA ligand was first incubated at pH 3–4 for 30 min at 95 °C followed by pH adjustment with 0.5 M MES buffer (pH 5.5), and subsequent incubation at pH 4–5 for an additional 30 min at 95 °C. Using the two-step strategy, they radiolabeled two therapeutic ligands (PSMA-617 and PSMA-I&T) with [^89^Zr]ZrCl_4_. The two-step method was utilized to increase the molar activity of the tracers for their imaging study. However, the two-step acidic radiolabeling condition did not work for the production of BCN-[^89^Zr]Zr-DOTA-GA in this current study.

**Scheme 2:**
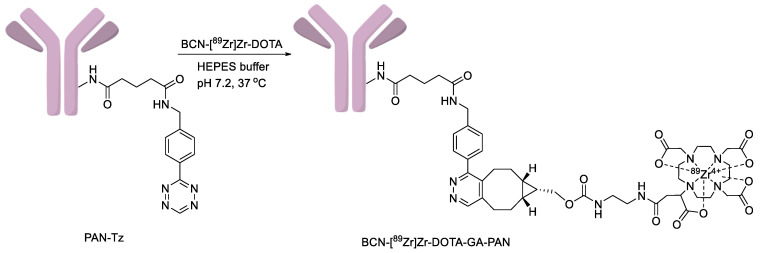
Preparation of BCN-[^89^Zr]Zr-DOTA-GA-PAN.

**Figure 3:**
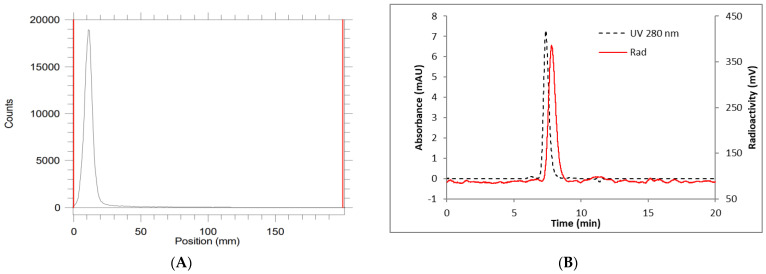
BCN-[^89^Zr]Zr-DOTA-GA-PAN; (**A**) radio TLC; (**B**) HPLC chromatogram of pure conjugates. TLC condition: plate, iTLC-SG, developer, 0.1 M EDTA pH 7; HPLC condition: column, TSKgel SuperSW3000 (4.6 mm ID × 30 cm, 4 µm); eluent, 0.1 M sodium phosphate, 0.1 M sodium sulfate, 0.05% sodium azide, 10% *iso*-propyl alcohol (pH 6.8), flow rate 0.35 mL/min; black line UV detector, red line radio detector. Retention time = 7.8 min.

**Figure 4:**
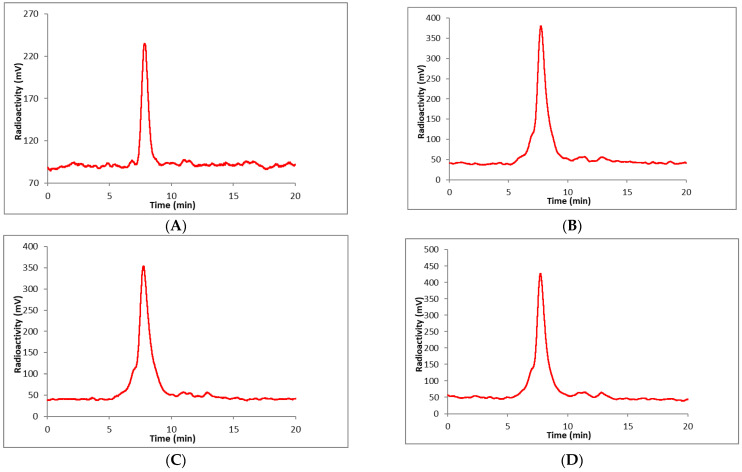
Radio HPLC chromatogram of BCN-[^89^Zr]Zr-DOTA-GA-PAN in whole human serum at 37 °C, (**A**) 0 min (after mixing with serum); (**B**) 3 d; (**C**) 5 d; (**D**) 7 d. HPLC condition: column, TSKgel SuperSW3000 (4.6 mm ID × 30 cm, 4 µm); eluent, 0.1 M sodium phosphate, 0.1 M sodium sulfate, 0.05% sodium azide, 10% *iso*propyl alcohol (pH 6.8), flow rate 0.35 mL/min. Retention time = 7.7 min.

The zirconium-89 labeling of the DOTA chelator requires elevated temperatures; therefore, it is not suitable for the direct labeling of heat-sensitive biomolecules like the antibody (PAN). Consequently, PAN radiolabeling must be performed through an indirect labeling approach. This method involves the initial preparation of labeled DOTA (BCN-[^89^Zr]Zr-DOTA-GA) followed by the coupling of this labeled prosthetic group to the antibody functionalized with tetrazene (PAN-Tz) via a copper-free cyclooctyne, tetrazine click reaction. To prepare the (PAN-Tz) conjugate, PAN was reacted with a 5-fold molar excess of the tetrazine NHS-active ester at pH 7.4 and purified by a PD-10 column using 0.5 M HEPES buffer (pH 7.2). The click reaction of BCN-[^89^Zr]Zr-DOTA-GA (Scheme 2) with PAN-Tz was monitored by radio TLC. The complete consumption of BCN-[^89^Zr]Zr-DOTA-GA was observed in 30 min at 37 °C with 1 mg of PAN-Tz. However, a reduced amount of IgG-Tz (0.5 mg) resulted in 70% consumption of BCN-[^89^Zr]ZrDOTA-GA. The final radiolabeled conjugate, BCN-[^89^Zr]Zr-DOTA-GA-PAN, was purified by a PD-10 column using saline (pH 7.0) as an eluent. The overall radiochemical yields starting from the radiolabeling of BCN-DOTA-GA were 40–50% (n = 12, decay uncorrected) in 2 h. The radiochemical purity assessed by TLC and HPLC was >95% (Figure 3). The molar activities were 200–300 Ci/mmol. The conjugate, BCN-[^89^Zr]Zr-DOTA-GA-PAN, was stable (95% intact after 7 d) in whole human serum at 37 °C up to 7 d (Figure 4). 

**Figure 5:**
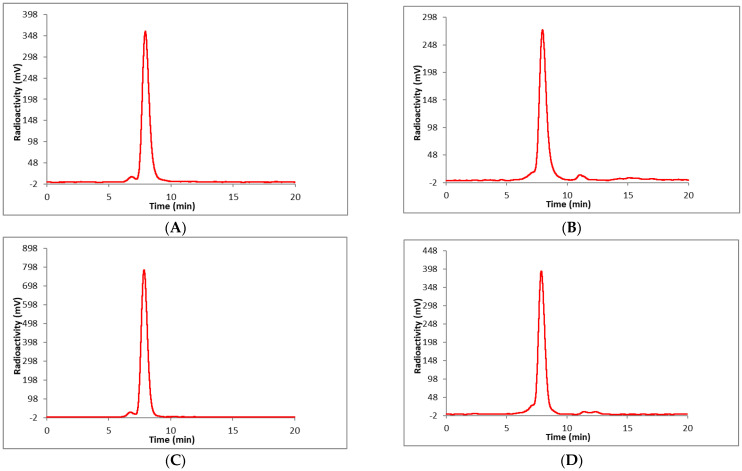
Radio HPLC chromatogram in whole human serum of [^89^Zr]Zr-DFO-PAN (**A**) 0 min (after mixing with serum); (**B**) 7 d and [^89^Zr]Zr-DFO*; (**C**) 0 min (after mixing with serum); (**D**) 7 d. HPLC condition: column, TSKgel SuperSW3000 (4.6 mm ID × 30 cm, 4 µm); eluent, 0.1 M sodium phosphate, 0.1 M sodium sulfate, 0.05% sodium azide, 10% *iso*propyl alcohol (pH 6.8), flow rate 0.35 mL/min. Retention time = 7.9 min ([^89^Zr]Zr-DFO-PAN) and 7.8 ([^89^Zr]Zr-DFO*).

To compare the stability of the BCN-[^89^Zr]Zr-DOTA-GA-PAN with DFO and DFO* conjugates, [^89^Zr]Zr-DFO-PAN and [^89^Zr]Zr-DFO*-PAN were prepared. The commercially available p-isothiocyanatobenzyl-desferrioxamine (DFO-Bz-NCS) is the most commonly used bifunctional chelator to prepare DFO conjugates. It is conjugated to primary amines of the antibody via thiourea linkage formation. The DFO*-Bz-NCS is also commercially available. For the current study, DFO-PAN and DFO*-PAN conjugates were prepared and purified following the literature method using a 5-fold molar excess of either DFO-Bz-NCS or DFO*-Bz-NCS [52]. The purities of the conjugates were determined by HPLC using a size-exclusion column (SE-HPLC). The concentrations of the conjugates were measured using the bicinchoninic acid (BCA) assay. The number of chelators per antibody (1.8 ± 0.2) was determined using 25 mM nonradioactive zirconium chloride mixed with a trace amount of zirconium-89 following the literature method [53]. Zirconium-89 labeling of the conjugates was performed according to the literature method to prepare [^89^Zr]Zr-DFO-PAN and [^89^Zr]Zr-DFO*-PAN [54]. The isolated radiochemical yields were in the range of 80–95% (n = 6). The molar activities of the conjugates were 1200–2700 Ci/mmol (n = 6) with a radiochemical purity >95% as confirmed by SE-HPLC. The stabilities of the conjugates were tested in whole human serum at various time points. Both conjugates were stable (95% intact after 7 d) in whole human serum at 37 °C up to 7 d (Figure 5). Although different linkers (Figure 1) were used for the formation of the radiolabeled PAN-DOTA and PAN-DFO/DFO* conjugates, comparable stabilities were observed in whole human serum at 37 °C. This result indicates that linkers do not have any significant impact on the stability of the labeled PAN conjugates (BCN-[^89^Zr]Zr-DOTA-GA-PAN, [^89^Zr]Zr-DFO-PAN, and [^89^Zr]Zr-DFO*-PAN).

**Figure 6:**
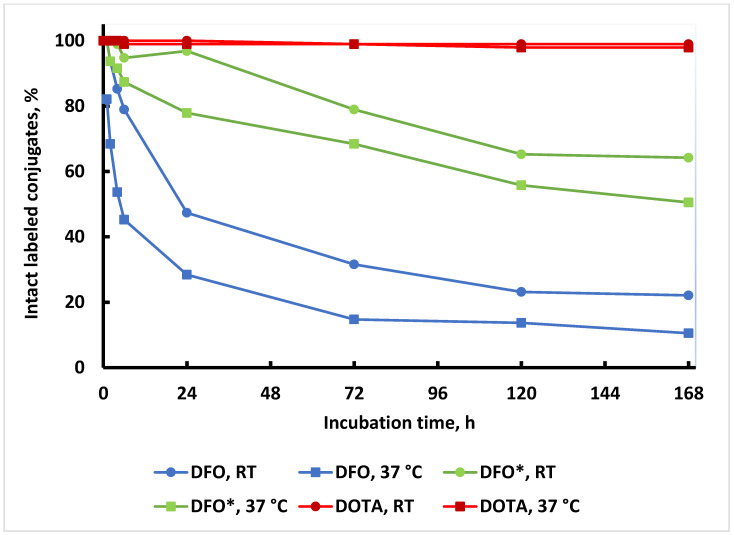
EDTA challenging study at room temperature and 37 °C of three conjugates: BCN-[^89^Zr]Zr-DOTA-GA-PAN (red), [^89^Zr]Zr-DFO-PAN (blue), and [^89^Zr]Zr-DFO*-PAN (green). The challenge reactions at room temperature are depicted as circles and at 37 °C as squares. The radiochemical purity of the conjugates was normalized to 100% at the start of the EDTA challenge.

The trans chelation experiments using EDTA as a challenging agent are the standard method for the determination of the kinetic inertness of zirconium-89 labeled conjugates [55]. Therefore, EDTA challenge reactions were performed for all three labeled conjugates with a large excess (1000 equivalents) of EDTA at room temperature and 37 °C up to 7 d (Figure 6). The degree of zirconium-89 trans chelation was determined by SE-HPLC at various time points. As expected and observed by others, rapid trans chelation was observed for the [^89^Zr]Zr-DFO-PAN conjugates at both temperatures (22% and 10% intact after 7 days at room temperature and 37 °C, respectively) [42,49]. Much slower trans chelation was observed for the [^89^Zr]Zr-DFO*-PAN (64% and 50% intact after 7 days at room temperature and 37 °C, respectively). In contrast, the BCN-[^89^Zr]Zr-DOTA-GA-PAN showed excellent stability against EDTA trans chelation at both temperatures (>94% intact at both temperatures).

## 3. Conclusions

Zirconium-89 labeling of the DOTA chelator was optimized through a two-step basic pH adjustment at 90 °C to prepare BCN-[^89^Zr]Zr-DOTA-GA. Using this labeled prosthetic group antibody conjugate, BCN-[^89^Zr]Zr-DOTA-GA-PAN was successfully prepared via click chemistry with high radiochemical yield and purity. All three conjugates demonstrated excellent stability in whole human serum. BCN-[^89^Zr]Zr-DOTA-GA-PAN maintained its inertness when exposed to a large excess of EDTA. However, a rapid trans chelation was observed for [^89^Zr]Zr-DFO-PAN, with the order of [^89^Zr]Zr-DFO-PAN > [^89^Zr]Zr-DFO*-PAN > BCN-[^89^Zr]Zr-DOTA-GA-PAN. This result suggests that the DFO conjugate exhibits the lowest chemical inertness, while the DOTA conjugate demonstrates the highest.

## 4. Materials and Methods

The p-isothiocyanatobenzyl-desferrioxamine (DFO-Bz-NCS) was purchased from Macrocyclics, Inc. (Plano, TX, USA). BCN-DOTA-GA was purchased from Chematech (France). DFO*-Bz-NCS was obtained from ABX GmbH (Radeberg, Germany). Panitumumab was acquired from NIH pharmacy (Bethesda, MD, USA). Sodium acetate and Tris-HCl were purchased from Thermo Fisher Scientific (Waltham, MA, USA). The lyophilized whole human serum was obtained from MP Biomedicals, LLC (Solon, OH, USA) and dissolved in 2 mL saline. This serum solution was directly used without inactivation for the stability studies. All other chemicals and reagents were purchased from Sigma Aldrich (St. Louis, MO, USA) and used without further purification. PD-10 desalting columns were obtained from GE Healthcare Biosciences (Pittsburgh, PA, USA). [^89^Zr]Zr-oxalate and zirconium-89 chloride were obtained from 3D Imaging (Little Rock, AR, USA). Analytical high-performance liquid chromatography (HPLC) analyses were performed on an Agilent 1200 Series instrument equipped with a multi-wavelength UV detector connected in series with a Bioscan flow count radio detector. The size-exclusion column, TSKgel SuperSW3000 (SE, 4.6 mm ID × 30 cm, 4 µm), was obtained from Tosoh Bioscience LLC. (King of Prussia, PA, USA). The iTLC papers were read in an Eckert & Ziegler TLC scanner (B-AR2000-1, Hopkinton, MA, USA). A bicinchoninic acid (BCA) Protein Assay Kit (Thermo Fisher Scientific) with bovine gamma globulin standard was used to determine the conjugate concentrations.

### 4.1. Synthesis of DFO-PAN and DFO*-PAN

The DFO and DFO* conjugates of panitumumab (PAN) were prepared according to the literature method [52]. Briefly, PAN (20 mg/mL) was buffer-exchanged into 0.1 M NaHCO_3_ (containing 0.9% NaCl, pH 8.9), the concentration was adjusted to 5 mg/mL using NaHCO_3_ (containing 0.9% NaCl, pH 8.9), and treated with a 5-fold molar excess of DFO-Bz-NCS or DFO*-Bz-NCS (5 mg/mL in DMSO). The mixture was gently rocked at 37 °C for 75 min before stopping the conjugation reaction with the addition of 1 M Tris (to a final concentration of 12–15 mM). The DFO-PAN and DFO*-PAN conjugates were purified twice by two PD-10 columns (spin protocol) into 0.25 M sodium acetate (pH 5.5).

### 4.2. Synthesis of [^89^Zr]Zr-DFO-PAN and [^89^Zr]Zr-DFO*-PAN

Zirconium-89 labeling of the conjugates (DFO-PAN and DFO*-PAN) was performed according to the literature method to obtain radiolabeled conjugates ([^89^Zr]Zr-DFO-PAN and [^89^Zr]Zr-DFO*-PAN) [54]. Briefly, a stock solution of [^89^Zr]Zr-oxalate (~440 MBq) was diluted with 1.2 mL of HEPES buffer (0.5 M, pH 7.1–7.3). From this stock solution, ~150 MBq of zirconium-89 was used per radiolabeling reaction. The aliquot of zirconium-89 (~150 MBq, 0.4 mL) was added to 20 µL of 2,5-dihydroxybenzoic acid (~5 mg/mL, pH adjusted to 7 with 2 M Na_2_CO_3_ solution). DFO or DFO* conjugate (0.6 mg) was added to the solution. The reaction mixture was further diluted with HEPES buffer (0.5 M, pH 7.1–7.3) to reach the final mixture volume of 1 mL. The reaction mixture was incubated for 1 h at room temperature and challenged with diethylenetriamine pentaacetate, DTPA, (0.1 M, 5 µL, pH 7) for an additional 10 min. The radiolabeled conjugates were purified by a PD-10 column using 0.9% NaCl (pH 7). The molar activity was determined by HPLC using a size-exclusion column (t_R_ = 8.0 min). The purity of the conjugate was determined by HPLC and TLC.

### 4.3. Synthesis of PAN-Tz

Panitumumab (PAN, 20 mg/mL, PBS buffer pH 7.4) was buffer-exchanged to 5 mg/mL and reacted with 5 eq excess of the tetrazine NHS ester (CAS#1244060-64-9, 5 mg/mL in DMSO) at 4 °C overnight. The conjugate was purified with 0.5 M HEPES buffer (pH 7.2) using a PD-10 column. The purity of the conjugate (PAN-Tz) was confirmed by analytical size-exclusion HPLC. The protein concentration was determined by BCA assay. The tetrazine to antibody ratio was 4.5 ± 0.2, as determined by Liquid Chromatography Electrospray Ionization Mass Spectrometry (LC-ESI-MS) using an Acquity UPLC H-Class coupled to a Xevo G2-XS QTof (Waters Corporation, Milford, MA, USA). The sample was de-glycosylated using Rapid PNGase F (New England BioLabs, Ipswich, MA, USA) before analysis and desalted online using an XBridge Protein BEH C4 column (Waters Corporation) with a water/acetonitrile gradient containing 0.1% formic acid. Data processing was performed using UNIFI software version 1.9.4.053 (Waters Corporation) and peaks were assigned based on the unmodified antibody as a reference.

### 4.4. Synthesis of BCN-[^89^Zr]Zr-DOTA-GA-PAN

HEPES buffer pH 7.2 (0.5 M; 445 µL) was added to the solution of [^89^Zr]Cl_4_ (115 µL 1N HCl). If the supplied volume of HCl is different, then the volume of HEPES buffer needs to be adjusted accordingly. An aliquot of this solution (130 µL, ~3 mCi) was added to the solution of BCN-DOTA-GA (15 µL, 30 µg, 43 nmol) in 0.5 M HEPES buffer (pH 7.4). The final pH of the solution was ~7.2. The reaction mixture was heated at 90 °C for 30 min followed by the addition of 135 µL 0.5 M HEPES (pH 7.68) and continued heating at the same temperature for an additional 30 min. The formation of the DOTA complex was checked by TLC and HPLC (TLC condition: iTLC-SG, developer, 0.1 M EDTA pH 7; HPLC condition: Agilent XDB C-18 (4.6 × 150 mm, 5µ); eluent, 0–45% B in 13 min, 45–90% B in 14 min, 90% B until 19 min, A = 0.05% TFA in water, B = 0.05% TFA in acetonitrile, flow rate 1 mL/min). PAN-Tz conjugate in 0.5 M HEPES buffer (1 mg, 4.8–5.2 mg/mL) was added and the reaction was incubated for 0.5 h at 37 °C. The radiolabeled conjugate was purified into 0.9% NaCl (pH 7) by a PD-10 column. The molar activity and the purity of the radiolabeled conjugate were determined by SE-HPLC (condition: TSKgel super3000 4.6 mm × 30 cm, 4 µm column; eluent: 0.1 M sodium phosphate, 0.1 M sodium sulfate, 0.05% NaN_3_: pH 6.8 + 10%IPA; flow rate: 0.35 mL/min). The stability of the radiolabeled conjugate in whole human serum at 37 °C was assessed by SE-HPLC and TLC for up to 7 d.

### 4.5. Storage and In Vitro Serum Stability

The storage stability of the conjugates was assessed in anticipation of any required storage before biological investigation. Following radiosynthesis, all three labeled conjugates ([^89^Zr]Zr-DFO-PAN, [^89^Zr]Zr-DFO*-PAN, and BCN-[^89^Zr]Zr-DOTA-GA-PAN) in saline were stored at 4 ^o^C for 24 h and reanalyzed by SE-HPLC to confirm the integrity of the antibody conjugates. All three conjugates were stable at those conditions for at least up to 24 h at 4 °C.

To determine in vitro serum stability, whole human serum (500 µL) was added to a solution of [^89^Zr]Zr-DFO-PAN, [^89^Zr]Zr-DFO*-PAN, or BCN-[^89^Zr]Zr-DOTA-GA-PAN (~500 µCi in 500 µL of saline, pH 7.0) and kept at 37 °C for up to 7 d. The radiochemical stability was determined at various time points by directly injecting an aliquot of the solution using the SE HPLC.

### 4.6. EDTA Challenge Study

To perform the EDTA challenge study, each labeled conjugate, BCN-[^89^Zr]Zr-DOTA-GA-PAN, [^89^Zr]Zr-DFO-PAN, and [^89^Zr]Zr-DFO*-PAN (200 µCi, in 250 µL saline, pH 7.0), was added to a solution of EDTA (1000 eq in HEPES buffer pH 7.0) and incubated at either room temperature or at 37 °C with gentle rocking for 7 days. The stabilities of the labeled conjugates were analyzed using SE-HPLC (size-exclusion column) by directly injecting an aliquot of the conjugates at 1 h, 2 h, 4 h, 6 h, 1 d, 3 d, 5 d, and 7 d.

## Figures and Tables

**Table 1:** Radiolabeling conditions tested to prepare BCN-[^89^Zr]Zr-DOTA-GA.

Entry ^a^	Amount of Ligand (µg)	Amount of Zirconium-89 (mCi)	Temperature (°C)	Yield (%) by Radio TLC
1	10	1	80	<20
2	10	1	90	<20
3	30	1	80	95
4	30	1	90	98
5	30	2	90	92
6	30	3	90	83
7	30	4	90	74

^a^ HEPES buffer (0.5 M), pH 7.2–7.6.

## Data Availability

Data is contained within the article.
